# Supplementary material for: Cryo-EM structure of the varicella-zoster virus A-capsid
Source: Nat Commun. 2020 Sep 22;11:4795. doi: 10.1038/s41467-020-18537-y (PMC7508878; doi:10.1038/s41467-020-18537-y)
Supplement: Supplementary file 1 — Supplementary Information [file 41467_2020_18537_MOESM1_ESM.pdf]

Supplementary Information

Cryo-EM structure of the varicella-zoster virus A-capsid

Sun et al.

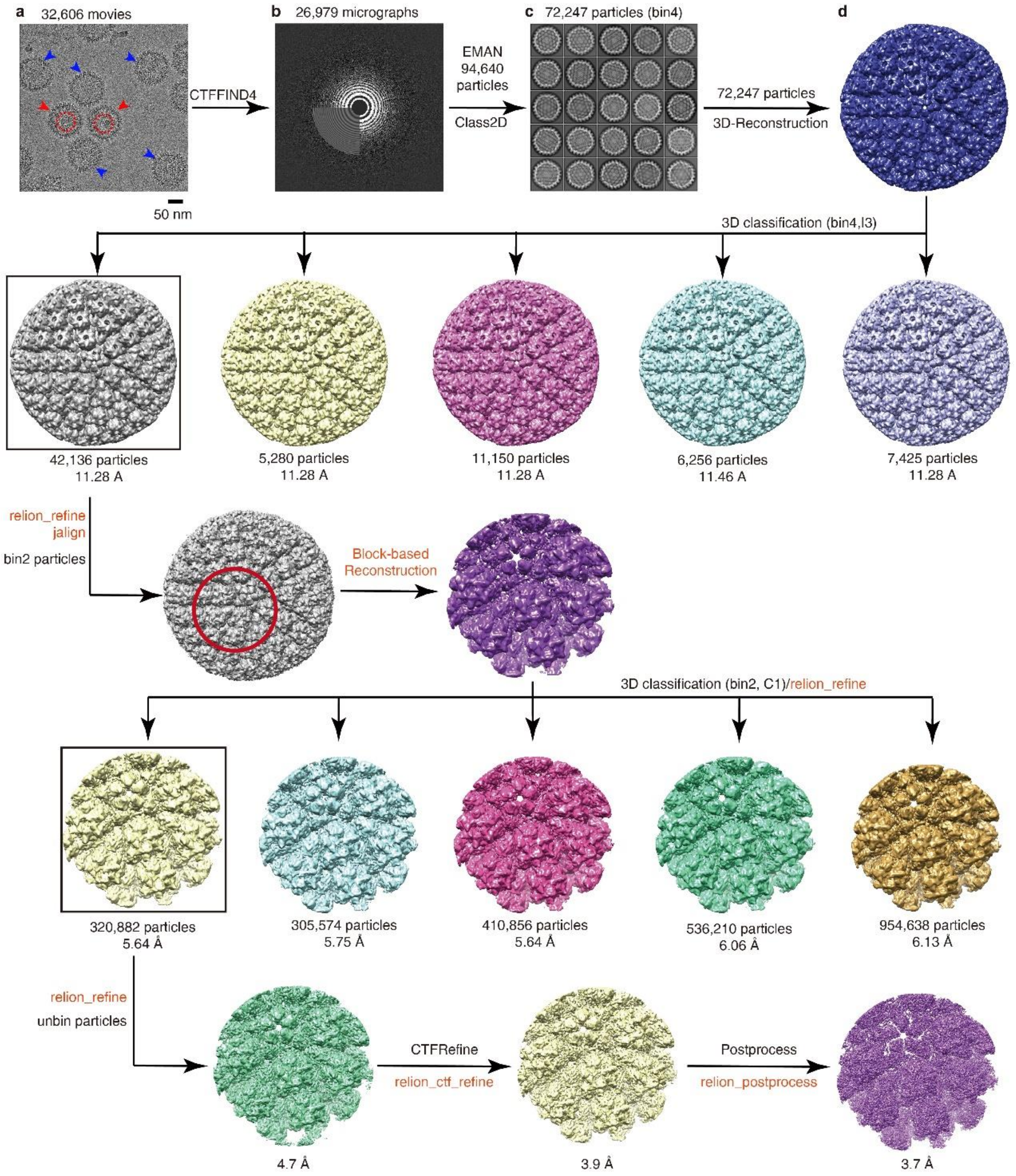

Supplementary Figure 1. Cryo-EM data processing of VZV capsid. a, A representative micrograph of the VZV capsid. Most of the particles are A-capsids (blue arrowheads) with a small proportion being B-capsids (red arrowheads). The inner density for the scaffold protein in a B-capsid is indicated by a dashed circle. **b,** Typical CTF image of a drift-corrected micrograph. **c,** Image gallery of 2D class averages. **d,** Workflow of 3D classification and reconstruction.

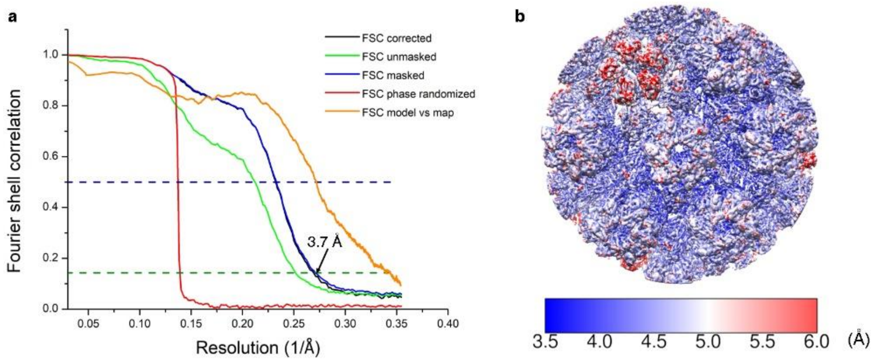

Supplementary Figure 2. Resolution assessment of the final reconstruction. a. The FSC curves for the final block-based reconstruction and model-map fit evaluation. **b,** Local resolution distribution of the density map generated by block reconstruction.

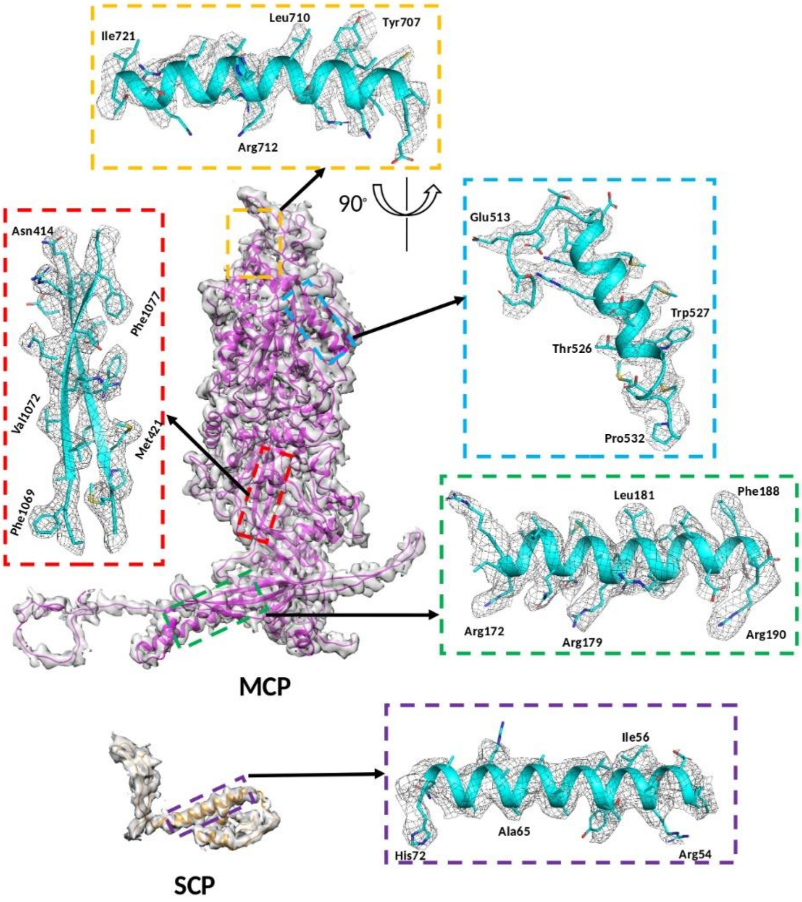

Supplementary Figure 3. Representative density and atomic models of the hexon MCP and SCP capsid proteins.

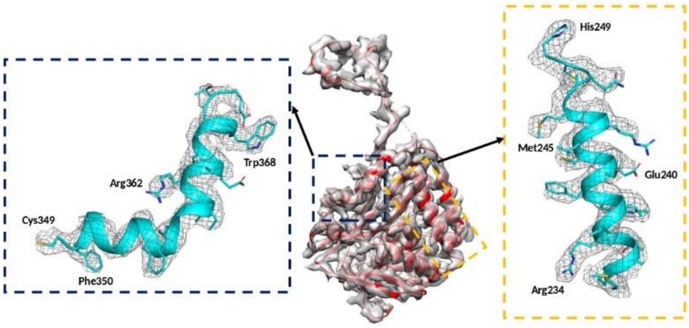

Tri1

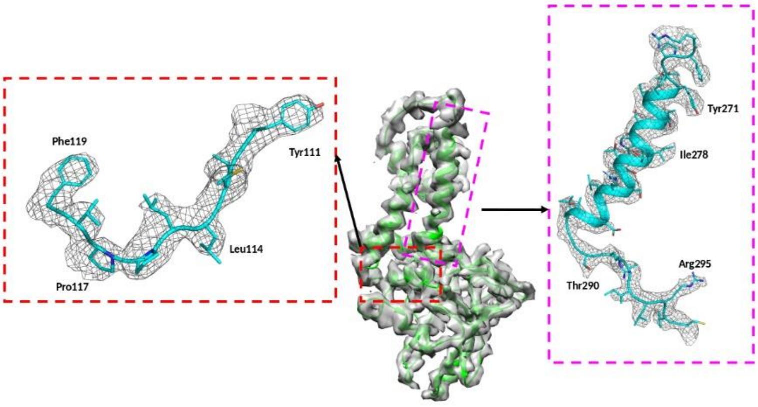

Tri2

Supplementary Figure 4. Representative density map and atomic models of the heterotriplex proteins.

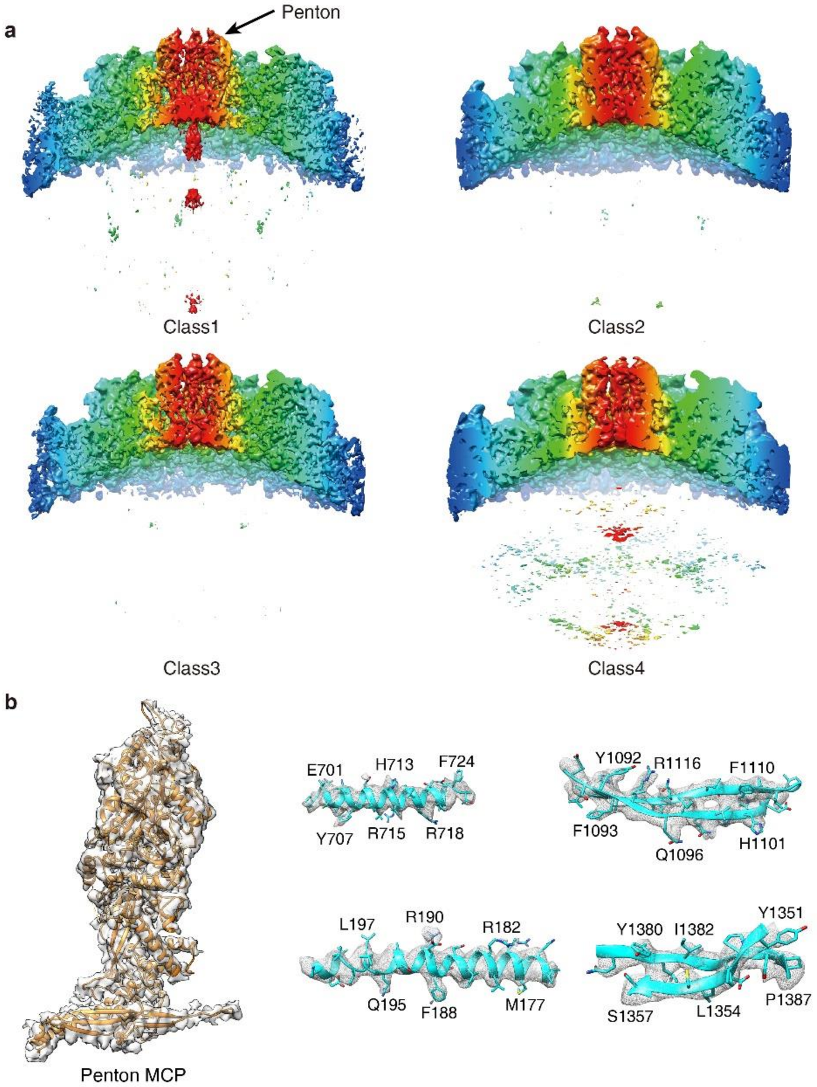

Supplementary Figure 5. The 3D classification for the 5-fold vertex sub-particles and representative density of the penton MCP. a, Density maps of different 3D classes for sub-particles of the vertexes. The maps are colored by radius from red to blue, centered at the 5-fold axis. **b,** Representative density map of the penton MCP.

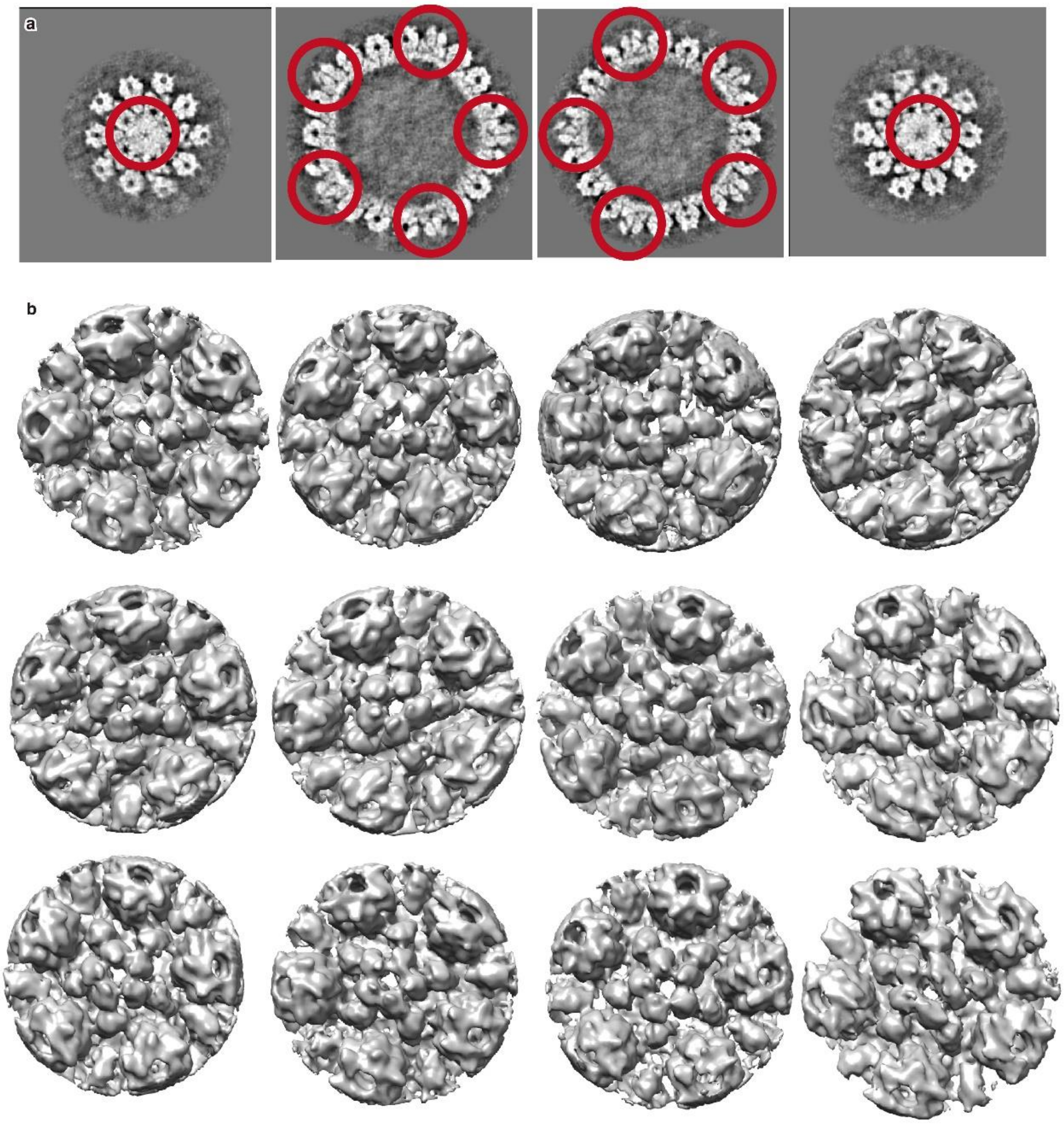

Supplementary Figure 6. The 3D classification of capsid particles with C1 symmetry. a, Four typical slices of the capsid density map showing the 12 vertexes, viewed along a 5-fold axis. The vertexes are highlighted with red circles. **b,** Close-up views of the density map for the 12 vertexes.

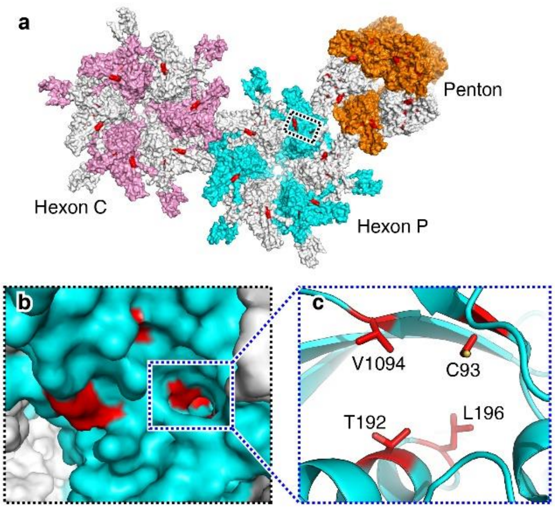

Supplementary Figure 7. Potential inhibitor-binding pocket beneath the floor of MCP. a, Overview of the location of the 35B2-binding pocket beneath the floor of the penton and hexon capsomers (viewed from inside of the capsid). The escape mutation residues are colored in red. **b–c,** Close-up view of the candidate inhibitor-binding pocket. The potential key contacting residues are shown as stick models.

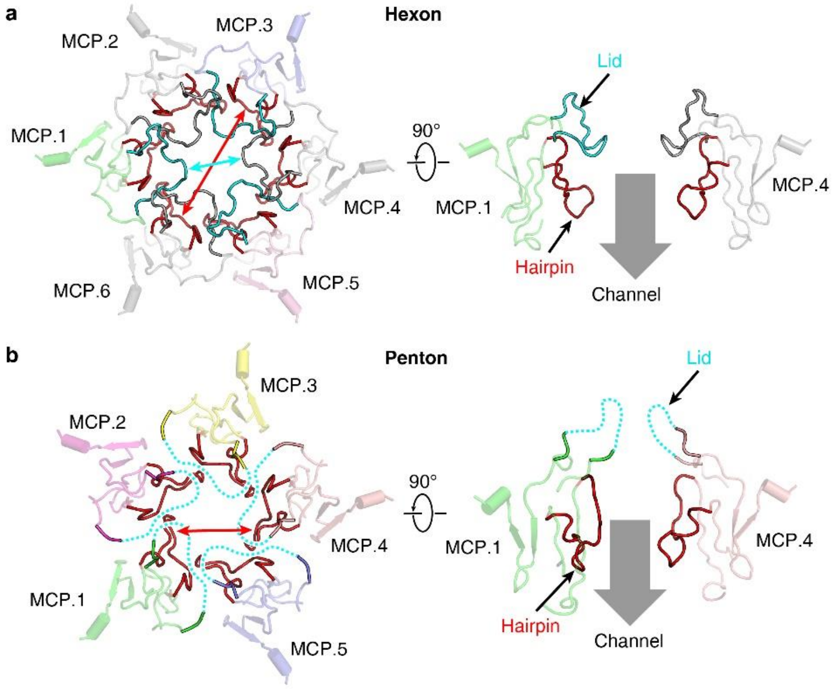

Supplementary Figure 8. Comparison of the central channel within penton and hexon capsomers. a, The channel in the hexon is formed by two motifs of MCP, a hairpin loop in the inner wall and a lid covering the top. The hairpin loops of 6 MCPs form a wide central channel in each hexon. **b,** The hairpin loops in the 5 MCPs of the penton are arranged with a narrow space in between. The lid loops are disordered in penton MCPs.

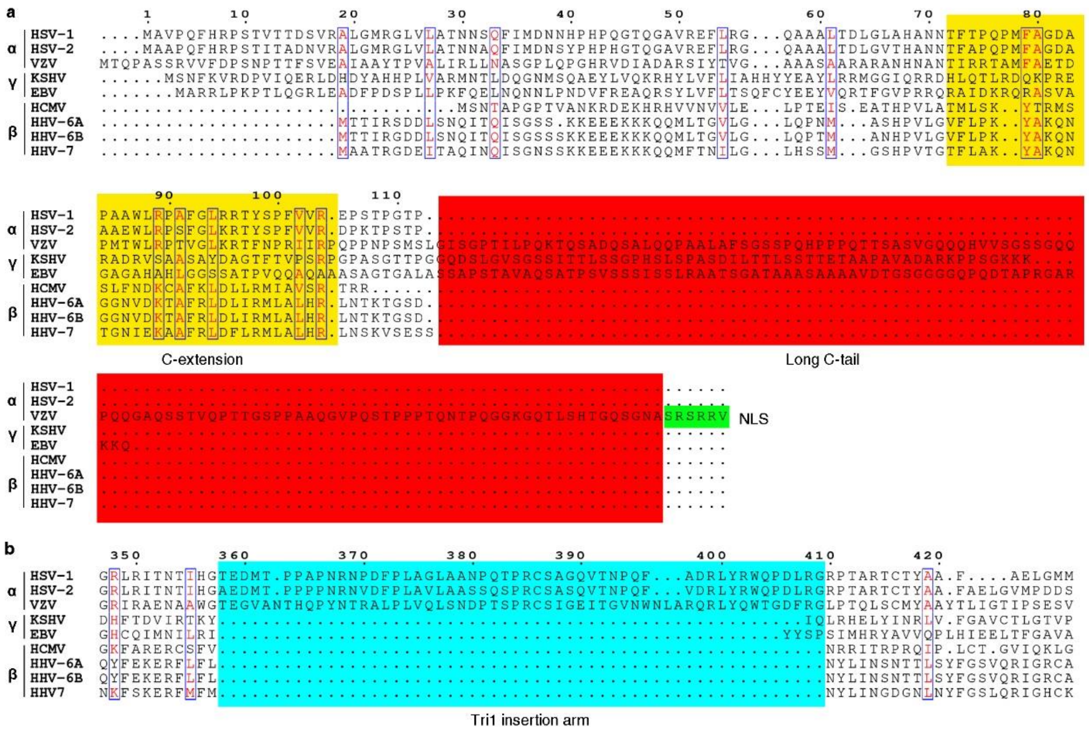

Supplementary Figure 9. Sequence alignment of SCP and Tri1 proteins in selected regions of nine human-infecting herpesviruses. a, Alignment of the SCP sequences. The C-extension region of the SCP is highlighted in yellow. The additional long C-tail of the VZV, EBV, and KSHV SCPs is indicated by red highlight. The unique C-terminal nuclear localization signal (NLS) of the VZV SCP is highlighted in green. **b,** Alignment of the Tri1 protein sequences. The internal insertion arm of Tri1 in the alphaherpesvirus capsid is highlighted in cyan. HSV-1, HSV-2, and VZV belong to the *Alphaherpesvirinae* subfamily; HCMV, HHV-6A, HHV-6B, and HHV-7 belong to the *Betaherpesvirinae* subfamily; and KSHV and EBV belong to the *Gammaherpesvirinae* subfamily.

Supplementary Table 1. Cryo-EM data processing and refinement statistics.

**Data Collection & Processing**	
Microscope	FEI Titan Krios
Camera	Falcon III
Magnification	59,000×
Voltage (kV)	300
Total dose ($e^{-}/Å^2$)	40
Defocus range (μm)	–0.8 to –1.5
Pixel size (Å/pixel)	1.41
Symmetry imposed	I3
Movies (total)	32,606
Initial particles images	94,640
Final particles images	42,136
Final block images	311,236
Block symmetry imposed	C1
Map resolution (Å, FSC = 0.143)	3.7
Map sharpening B-factor ($Å^2$)	–158.691
Map resolution range (Å, FSC = 0.143)	3.7
**Refinement**	
Initial model used (PDB code)	5ZZ8
Non-hydrogen atoms	208,865
Protein residues	26942
Ligands	0
Lipids	0
Ions	0
Validation	
Clash score	7.93
Poor rotamers (%)	0.87
Root-mean-square deviations	
Bond length (Å)	0.003
Bond angle (°)	0.576
Ramachandran statistics (%)	
Most favored	91.51
Allowed	8.40
Outliers	0.09

Supplementary Table 2. Genome size of different human herpesviruses.

α	HHV-1 (HSV-1)	152,222 bp
	HHV-2 (HSV-2)	154,675 bp
	HHV-3 (VZV)	124,884 bp
β	HHV-5 (HCMV)	235,403 bp
	HHV-6A	159,378 bp
	HHV-6B	161,573 bp
	HHV-7	153,080 bp
γ	HHV-4 (EBV)	172,764 bp
	HHV-8 (KSHV)	137,969 bp
